# Lithium Chloride Inhibits Vascular Smooth Muscle Cell Proliferation and Migration and Alleviates Injury-Induced Neointimal Hyperplasia via Induction of PGC-1α

**DOI:** 10.1371/journal.pone.0055471

**Published:** 2013-01-31

**Authors:** Zhuyao Wang, Xiwen Zhang, Siyu Chen, Danfeng Wang, Jun Wu, Tingming Liang, Chang Liu

**Affiliations:** 1 Jiangsu Key Laboratory for Molecular and Medical Biotechnology and College of Life Sciences, Nanjing Normal University, Nanjing, Jiangsu, China; 2 Department of Cardiology, Huai'an First People's Hospital, Nanjing Medical University, Huai'an, Jiangsu, China; 3 Department of Cardiology, The First Affiliated Hospital of Nanjing Medical University, Nanjing, Jiangsu, China; William Harvey Research Institute, Barts and The London School of Medicine and Dentistry, Queen Mary University of London, United Kingdom

## Abstract

The proliferation and migration of vascular smooth muscle cells (VSMCs) contributes importantly to the development of in-stent restenosis. Lithium has recently been shown to have beneficial effects on the cardiovascular system, but its actions in VSMCs and the direct molecular target responsible for its action remains unknown. On the other hand, PGC-1α is a transcriptional coactivator which negatively regulates the pathological activation of VSMCs. Therefore, the purpose of the present study is to determine if lithium chloride (LiCl) retards VSMC proliferation and migration and if PGC-1α mediates the effects of lithium on VSMCs. We found that pretreatment of LiCl increased PGC-1α protein expression and nuclear translocation in a dose-dependent manner. MTT and EdU incorporation assays indicated that LiCl inhibited serum-induced VSMC proliferation. Similarly, deceleration of VSMC migration was confirmed by wound healing and transwell assays. LiCl also suppressed ROS generation and cell cycle progression. At the molecular level, LiCl reduced the protein expression levels or phosphorylation of key regulators involved in the cell cycle re-entry, adhesion, inflammation and motility. In addition, *in vivo* administration of LiCl alleviated the pathophysiological changes in balloon injury-induced neointima hyperplasia. More importantly, knockdown of PGC-1α by siRNA significantly attenuated the beneficial effects of LiCl on VSMCs both *in vitro* and *in vivo*. Taken together, our results suggest that LiCl has great potentials in the prevention and treatment of cardiovascular diseases related to VSMC abnormal proliferation and migration. In addition, PGC-1α may serve as a promising drug target to regulate cardiovascular physiological homeostasis.

## Introduction

The proliferation and migration of vascular smooth muscle cells (VSMCs) is the major underlying biological process of pathological conditions such as in-stent restenosis. When the endothelial injury is induced by balloon catheterization or stent placement, it will stimulate VSMCs to migrate from the medial and the adventitial layers to the intimal layer of the vessel wall where they proliferate [1]. The formation of this neointima is an important architectural change in the vessel wall that leads to restenosis after angioplasty or stenting [2]. The accelerated neointima formation and the increased rates of restenosis are observed in patients with metabolic disorders such as diabetes [3], [4]. At the molecular level, mechanical injury to the vessel wall initiates the release of growth factors, which subsequently activate VSMCs through various key signaling pathways including the MAPK cascade [5], [6]. Re-entry of quiescent VSMCs into the cell cycle is a hallmark molecular event in the restenosis process [7]. Therefore, a strategy aiming to inhibit VSMC growth and migration has high value in the prevention and attenuation of in-stent restenosis upon percutaneous transluminal coronary angioplasty (PTCA).

Peroxisome proliferator-activated receptor γ coactivator-1α (PGC-1α) is a transcriptional coactivator intensively involved in the regulation of cellular energy metabolism [8]. As is ubiquitously expressed in tissues with high energy consumption levels, PGC-1α stimulates various physiological processes such as mitochondrial biogenesis, hepatic fatty acid β-oxidation, muscle fibre switch and circadian clock machinery by selectively activating nuclear receptors and transcriptional factors. Specifically, recent studies have disclosed the roles of PGC-1α in VSMC physiological homeostasis. For example, several mitogenic stimuli promote VSMC proliferation and migration while decrease PGC-1α expression [9]–[11]. In contrast, restoration of PGC-1α levels by using forced adenovirus transduction or hormone administration inhibits overactivated VSMC growth and migration. PGC-1α also attenuates TNF-α-induced inflammation and oxidative stress in VSMCs [12]. *In vivo* studies indicated that overexpression of PGC-1α suppresses neointimal formation in the injured rat carotid artery [13]. Collectively, PGC-1α negatively regulates the pathological activation of VSMCs and increase of PGC-1α expression may have great potentials to treat restenosis.

As one of the lightest solid elements, lithium has been used as a mood stabilizer for more than one century and it was the first drug approved by FDA in 1974 for maintenance treatment of bipolar disorder [14]. Mechanistical studies demonstrated that lithium specifically inhibits glycogen synthase kinase-3β (GSK-3β), as well as the enzymes involved in the inositol phosphate metabolic process [15]. These events will subsequently activate Wnt/β-catenin signal pathway and will lead to the clearance of the cytosol inositol accumulation. Notably, the protective effects of lithium on cardiovascular system have recently been revealed. For example, lithium chloride (LiCl) down-regulates vascular cell adhesion molecule-1 (VCAM-1) expression in human umbilical vein endothelial cells and improves atherosclerotic lesion in high-fat diet fed ApoE-deficient mice [16]. Furthermore, LiCl selectively induces apoptosis in macrophages and stabilizes atherosclerosis plaque [17]. But to date, these previous studies stay in the descriptive stage and the direct molecular target responsible for the beneficial action of lithium in cardiovascular system remains unknown. Given that lithium increases PGC-1α expression in endothelial cells [18], we carried out the present study to investigate that whether lithium inhibits VSMC proliferation and migration by similarly upregulating PGC-1α levels.

## Results

### Increase of PGC-1α protein expression and nuclear localization by LiCl

**Figure 1:**
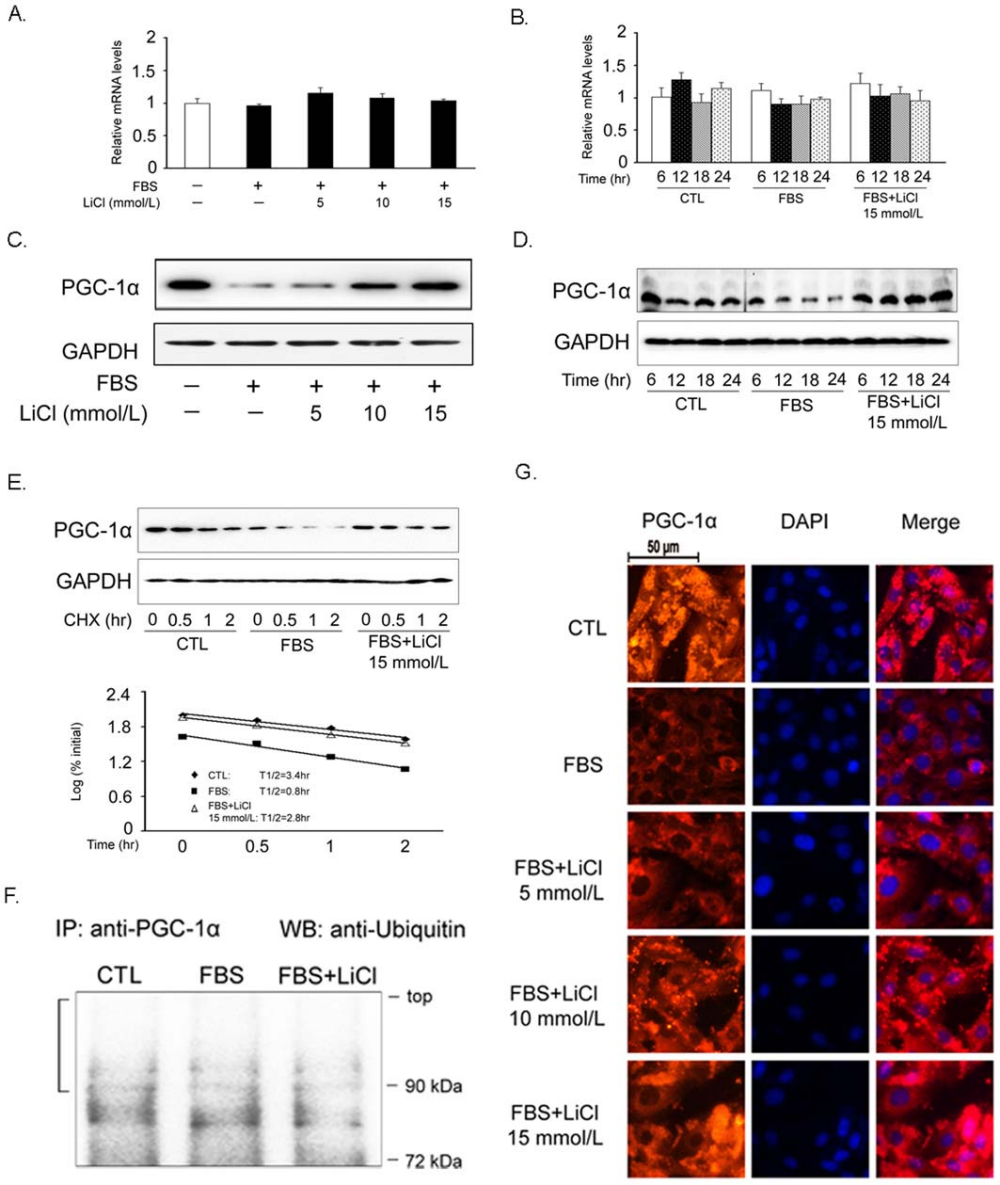
LiCl increases PGC-1α protein expression in VSMCs. Cells were pretreated with 15 mmol/L LiCl for 30 min and then stimulated with FBS for 24 hr unless otherwise indicated. The mRNA and protein expression levels of PGC-1α were determined by RT-qPCR (A, B) and Western blot (C, D), respectively. PGC-1α protein levels in a cycloheximide chase experiment (E). To determine the half-life of PGC-1α protein, the pixels for each band were measured and normalized so that the number of pixels at *t* = 0 was 100%. The log_10_ of the percent of pixels was plotted *versus* time for each time point, and the *t*1/2 was calculated from the log of 50%. PGC-1α protein ubiquitination determined by coimmunoprecipitation assay (F). The cellular localization of PGC-1α was shown by immunocytochemistry (G). The red and blue area represents PGC-1α protein and DAPI-stained nuclei. Scale bar indicates 50 µm. CTL: control, cells were treated with 15 mmol/L NaCl. Data are reported as means ± SEM from three independent experiments.

We firstly assessed the effects of LiCl on PGC-1α expression in VSMCs. As demonstrated in Fig. 1A and 1B, neither LiCl nor FBS altered PGC-1α expression at the transcriptional level. However, FBS suppressed PGC-1α protein expression level, which could be reversed by LiCl pretreatment in a dose-dependent manner (Fig. 1C). As incubation time extended, LiCl gradually increased PGC-1α protein expression, suggesting that such induction was also time-dependent (Fig. 1D). To exclude the possibility that PGC-1α upregulation by LiCl is specific for FBS stimulation, we challenged VSMCs with other mitogens such as oleci acid and PDGF-BB and observed the similar trends (Fig. S1). To test the hypothesis that increased PGC-1α protein accumulation induced by LiCl was due to enhanced protein stability, the turnover of PGC-1α protein was investigated with a CHX chase experiment. As shown in Fig. 1E, PGC-1α protein stability was not increased in LiCl-treated VSMCs. The half-life of PGC-1α protein was 3.4 hr, 0.8 hr, and 2.8 hr for the control, FBS-treated, and FBS plus LiCl-treated VSMCs, respectively. Consistent with this finding, the ubiquitination of PGC-1α protein was comparable with that of control cells (Fig. 1F). In addition to the accelerated synthesis of protein expression, the nuclear localization of PGC-1α became more robust, indicating that this transcriptional coactivator was activated by LiCl in our settings (Fig. 1G).

### Suppression of VSMC proliferation and migration by LiCl

**Figure 2:**
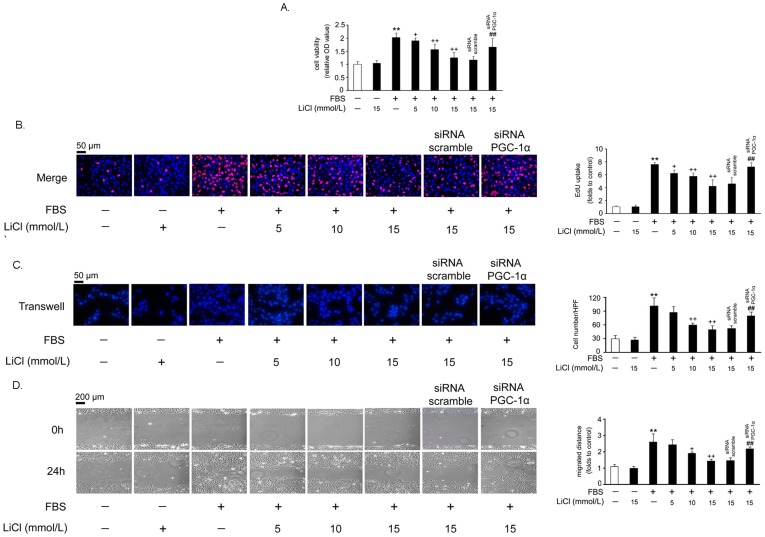
LiCl attenuates FBS-induced VSMC proliferation and migration. VSMCs were infected with recombinant adenoviruses expressing PGC-1α siRNA or scrambled siRNA for 30 hr and then treated as previously described in Fig. 1. Cell proliferation was evaluated by MTT (A) and Edu (B) assays. The scale bar indicates 50 µm. For the determination of cell migration, transwell (C) and wound healing (D) assays were used. Migrated cells were quantified by the average of 5 randomly chosen high-power fields (HPF) of 3 independent duplicate experiments. The scale bar indicates 50 µm (C) or 200 µm (D). **P<0.01 compared with the control group; ^+^ *P*<0.05, ^++^ *P*<0.01 compared with the serum-treated group; ^##^ *P*<0.01 compared with the control siRNA group.

MTT assay showed that LiCl alone did not affect the basal level of VSMC proliferation (Fig. 2A). However, LiCl (5, 10 or 15 mmol/L) dose-dependently inhibited FBS-induced VSMC proliferation, and 15 mmol/L LiCl showed the strongest inhibitory effect (*P*<0.01 versus FBS) (Fig. 2A). To explore whether these effects of LiCl were dependent on the PGC-1α induction, we knocked down PGC-1α expression by siRNA adenovirus infection (Fig. S2) and found that the block of VSMC proliferation by LiCl was partially released (Fig. 2A). The results from MTT assay were confirmed by EdU incorporation assay (Fig. 2B, the pink dots represent EdU-incorporated nuclei).

Secondly, we used transwell assay to examine the effect of LiCl on quiescent and FBS-stimulated VSMC migration. In Fig. 2C, LiCl also showed no effect on quiescent VSMC migration. However, FBS-induced VSMC migration was inhibited by LiCl (*P*<0.01 versus FBS). Similarly, knock down of PGC-1α partially blocked the inhibitory effects of LiCl on VSMC migration. Directional migration of VSMC monolayers after mechanical wounding was performed next, and the data presented in Fig. 2D confirmed the results of the transwell assay.

### Suppression of ROS generation by LiCl

**Figure 3:**
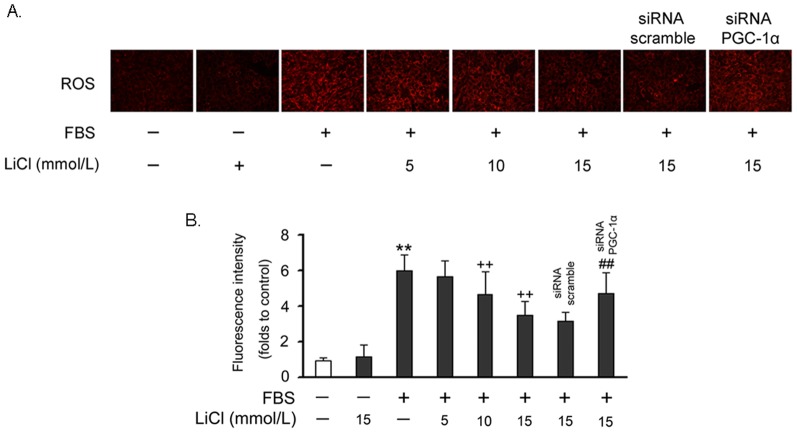
LiCl reduced ROS generation in VSMCs. Cells were pretreated with 15 mmol/L LiCl for 24 hr and then incubated with FBS for 30 min. ROS generation was measured using MitoTracker Red probe (CM-H2XRos). (A) A representative image was shown from three separate experiments. (B) Quantified data in each experimental group. The data of three independent experiments are expressed as the fold increase compared with control. **P<0.01 compared with the control group; ^++^ *P*<0.01 compared with the serum-treated group; ^##^ *P*<0.01 compared with the control siRNA group.

To directly evaluate the effect of LiCl on ROS production, we quantified ROS levels in FBS-treated VSMCs using MitoTracker staining. As shown in Fig. 3A and 3B, treatment with FBS for 30 min caused a greater increase of red fluorescence compared with control cells. However, pretreatment with LiCl for 24 hr significantly inhibited ROS generation and knock down of PGC-1α attenuated such inhibition.

### Retard of cell cycle progression by LiCl

**Figure 4:**
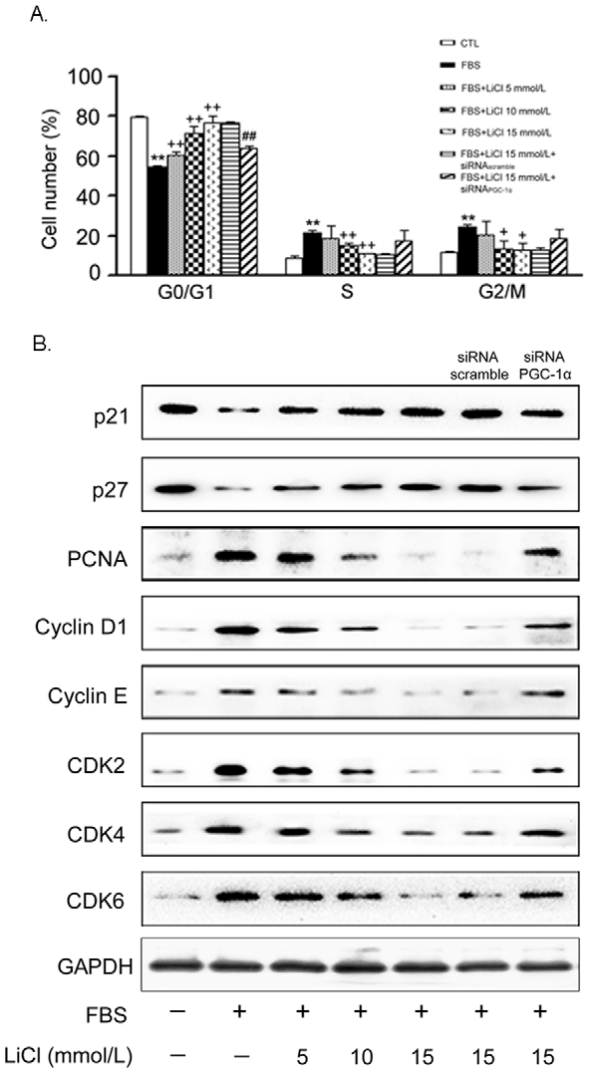
LiCl retards cell cycle progression. Cells were treated as previously described in Fig. 2. Cell cycle progression was assessed by FACS analysis (A). Data are presented as means ± SEM from three independent experiments. The protein levels of key regulators involved in the cell cycle progression were determined by Western blot (B). GAPDH was used as an internal control. A representative image was shown from three separate experiments. CTL: control, cells were treated with 15 mmol/L NaCl.

Since cell cycle progression is tightly associated with accelerated cellular proliferation, we subsequently used flow cytometry to investigate how LiCl affects cell cycle phases. As shown in Fig. 4A, FBS significantly increased the percentage of S-phase cells. In contrast, administration of LiCl antagonized the effects of FBS and caused more cells arrested in the G_0_/G_1_ phase. Once again, knock down of PGC-1α partially released this block. These data suggest that the inhibition of VSMC proliferation by LiCl may be due to the PGC-1α-mediated cell cycle arrest.

At the molecular level, LiCl reduced the protein expression level of proliferating cell nuclear antigen (PCNA), verifying its inhibitory effects on VSMC proliferation (Fig. 4B). On the other hand, cell cycle progression is controlled by cyclins and cyclin-dependent kinases (CDKs). We found that the FBS-induced protein expression levels of cyclin D1, cyclin E, as well as CDK2/4/6, were all suppressed by LiCl in a concentration-dependent manner. In contrast, p21 and p27 are negative regulators of the cyclin-CDK complexes, and their protein expression was correspondingly induced by LiCl (Fig. 4B). All these changes were partially abolished by knock down of PGC-1α.

### Inhibition of adhesion molecule expression by LiCl

**Figure 5:**
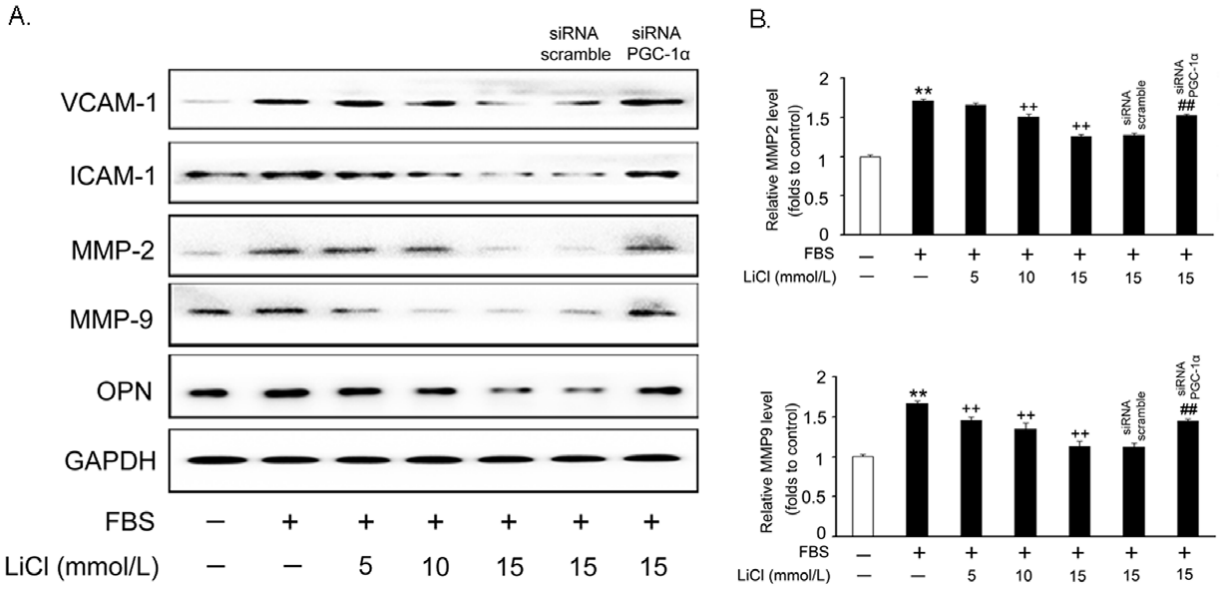
LiCl inhibits FBS-induced adhesion molecule expression. VSMCs were treated as previously described in Fig. 2 and Western blot was used to measure the protein levels involved in the cell adhesion and migration (A). The concentrations of MMP-2 and -9 in the culture medium was detected by ELISA (B). Data are presented as means ± SEM from three independent experiments.

Intercellular adhesion molecule 1 (ICAM-1), vascular cell adhesion molecule 1 (VCAM-1), matrix metalloproteases (MMPs) and osteopontin (OPN) are key regulators involved in cell migration. As shown in Fig. 5A, LiCl pretreatment inhibited the upregulation of all these proteins by FBS in a dose-dependent manner. Consistent with the decrease in MMP-2 and MMP-9 protein levels, ELISA assay showed that LiCl decreased the concentration of MMP-2 and MMP-9 released to the medium by 70% and 60%, respectively, compared with that induced by FBS alone (Fig. 5B). These findings suggest that LiCl inhibits the VSMC migration induced by FBS via suppressing the expression of migration-related proteins in these cells.

### Blockade of VEGF signaling cascade by LiCl

**Figure 6:**
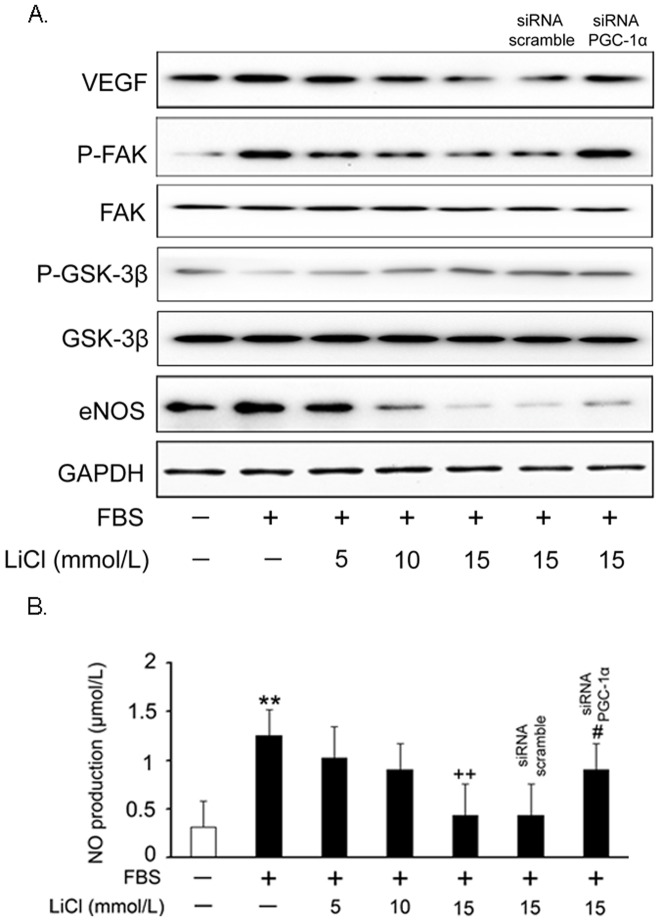
LiCl blocks FBS-induced activation of signaling cascades. VSMCs were treated as previously described in Fig. 2 and the cell lysates were subjected to Western Blot analysis (A). Note: for the detection of kinase phosphorylation, FBS stimulation time was shortened to 30 min. Measurement of NO production (B). Data are presented as means ± SEM from three independent experiments. **P<0.01 compared with the control group; ^++^ *P*<0.01 compared with the serum-treated group; ^#^ *P*<0.05 compared with the control siRNA group.

To further delineate the cellular and molecular mechanisms underlying LiCl-induced VSMC growth and migration inhibition, we evaluated the effect of LiCl on the vascular endothelial growth factor (VEGF) signaling cascades. After pretreatment with LiCl, VSMCs were stimulated with FBS and the VEGF and endothelial nitric oxide synthase (eNOS) protein expression levels, as well as the phosphorylation status of focal adhesion kinase (FAK), were measured by Western blot analysis using specific antibodies. As shown in Fig. 6A, LiCl markedly inhibited VEGF signaling activation by FBS in a dose-dependent manner. Furthermore, the phosphorylation level of GSK-3β was increased by LiCl, suggesting that its activation was blocked as expected. However, PGC-1α knockdown did not rescue the activity of GSK-3β. Consistent with these findings, the NO production was also reduced by LiCl (Fig. 6B). In addition, the inhibitory effects of LiCl on VEGF signaling cascade were attenuated when PGC-1α was knocked down.

### Inhibition of neointimal hyperplasia by LiCl

To investigate the role of LiCl in regulating VSMC proliferation *in vivo*, rat blood and carotid arteries were harvested on day 15 after balloon injury. Serological analysis demonstrated that in the LiCl-treated group, the serum levels of hepatic injury hallmarks, including ALT, AST, ALP, GGT and LDH, remained at the basal levels compared with the control group, suggesting that the dose of LiCl we selected was safe to the rat health (Table 1).

**Table 1:** Plasma parameters in male Sprague-Dawley rats 15 days after balloon injury *vs.* sham rats (n = 6).

Variable	Sham	Injury	Injury+LiCl
ALT (U/L)	54.3±1.23	54±0.70	48.9±0.75
AST (U/L)	172.1±1.23	180.9±0.70	206.3±0.75
ALP (U/L)	115.5±25.31	134.1±5.37	113.55±20.15
GGT (U/L)	2.45±0.50	3.9±0.14*	2.6±0.85
LDH (U/L)	1643.5±78.49	1696±193.75	1806±65.05
Plasma Li^+^ (mmol/L)	___	___	0.08±0.01

Values are mean ± SEM. Note: all the plasma parameters measured in injured or injured+LiCl group did not change significantly when compared with the sham group except GGT.

* *P*<0.05 compared with the sham group. ALT, alanine aminotranferease; AST, aspartate aminotransferase; ALP, alkaline phosphatase; GGT, γ-glutamyltransferase; LDH, lactate dehydrogenase.

**Figure 7:**
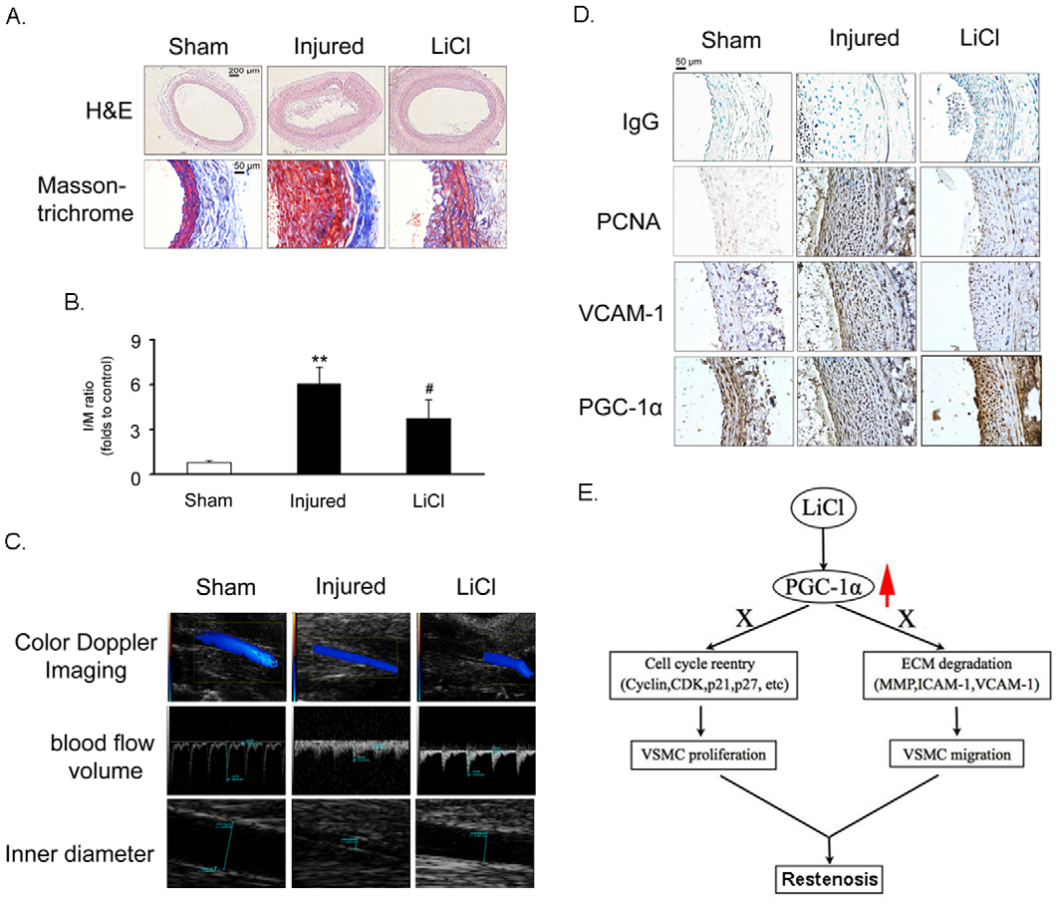
LiCl alleviates neointimal hyperplasia. The sections of rat carotid arteries were prepared on day 15 after balloon injury. (A) H & E staining and Masson-Goldner trichrome staining (below). The scale bar indicates 200 µm (upper) or 50 µm (below). (B) I/M thickness ratio analysis. **P<0.01 compared with the sham group, ^#^ *P*<0.05 compared with injured group (n = 6). (C) Doppler ultrasound examination. (D) Immunohistochemistry analysis. The scale bar indicates 50 µm. Non-immune IgG was used as a negative control. (E) A model for the regulation of VSMC proliferation and migration by LiCl through PGC-1α.

As expected, balloon injury led to an increased neointima area, and LiCl (10 mg/kg/day) significantly alleviated neointimal hyperplasia compared with injured controls (Fig. 7A). Masson & trichrome staining indicated that less collagen fibers (blue) and muscle fibers (red) accumulated in carotid arteries of LiCl-treated rats (Fig. 7A). Coincided with these findings, the I/M ratio index was increased in the injury group, which was partially reversed by LiCl treatment (Fig. 7B). Furthermore, doppler ultrasound examination demonstrated that blood flow volume was decreased accompanied with the narrowed inner diameter of carotid arteries in injured group. However, these pathophysiological changes were markedly improved by LiCl (Fig. 7C). Finally, immunohistochemical analysis revealed that LiCl inhibited the injury-induced increase in PCNA and VCAM-1 expression in neointima of carotid arteries, but up-regulated PGC-1α expression (Fig. 7D). These results suggest that LiCl exhibits an inhibitory effect on cell proliferation *in vivo*, and may be an effective agent for prevention of restenosis after angioplasty.

To further dissect the role of PGC-1α in mediating the effects of LiCl on VSMC layer in *vivo*, we delivered PGC-1α siRNA to balloon-injured rat carotid arteries through transfection. As shown in Fig. S3, injury indeed decreased PGC-1α protein expression, which was restored by LiCl treatment. PGC-1α siRNA delivery successfully blocked LiCl-induced PGC-1α up-regulation as compared with scramble siRNA. Coincided with the *in vitro* data, while injury de-phosphorylated GSK-3β, LiCl increased the phosphorylation level of this kinase. However, PGC-1α knockdown did not show any obvious effect on the phosphorylation of GSK-3β. With PGC-1α siRNA knockdown, the injury-induced neointimal hyperplasia of arteries was again worsened even when LiCl treatment was present, compared with scramble siRNA knockdown (Fig. S4A). Accordingly, the ratio of neointima to media area was significantly higher (2.2-fold) in PGC-1α siRNA- than scramble siRNA-treated arteries at 14 days (Fig. S4B). In contrast, the media area and circumference of external elastic lamina did not significantly differ with the two treatments.

## Discussion

Although pharmaceutical companies have spent great efforts in developing novel discovery technologies, such as structure-based drug design, combinatorial chemistry, high-throughput screening and genomics, the output is far less than the enormous increases in pharma R&D spending and this has become a severe problem for the biopharmaceutical industry [19]. Such productivity problem, together with the worldwide pressure on prices, has forced many drug developers to become more creative in finding new uses for, and improved versions of, existing drugs [20]. This drug development strategy is now called as “drug repositioning” and offers many advantages such as reduced risk, lower economic cost and the shorter routes to the clinic [21]. This is because repositioning candidates usually have undergone several stages of drug development including *in vitro* and *in vivo* screening, chemical optimization, toxicology, bulk manufacturing, formulation development and even early clinical evaluation and therefore have well-known safety and pharmacokinetic profiles. As such, repositioning can offer a better approach for the drug discovery.

As an old drug, lithium has been used to treat bipolar disorder for a long time. Recently, the additive effects of lithium on cardiovascular system have attracted much attention. Based on the previous reports, we found that LiCl increases PGC-1α protein expression and nuclear translocation in VSMCs (Fig. 1), while inhibits serum-induced cell proliferation and migration (Fig. 2), ROS generation (Fig. 3) and cell cycle re-entry (Fig. 4). At the molecular level, LiCl reduces the expression of key regulators involved in the cell cycle, migration, inflammation and adhesion (Fig. 5). The activation of critical kinases acting in VSMC pathological changes is also suppressed by LiCl (Fig. 6). More importantly, all these beneficial effects of LiCl on VSMCs are significantly attenuated by PGC-1α knockdown, strongly suggesting that PGC-1α is an essential mediator. Finally, LiCl inhibits neointimal formation, accompanied with reduction of cell proliferation after vascular injury in rats (Fig. 7). These results substantiate our *in vitro* findings and support the notion that LiCl confers protection against injury-induced pathological vascular remodeling. Our data provide evidences for the repositioning of lithium and clearly suggest that LiCl may be considered as a promising drug for the prevention and treatment of cardiovascular diseases related to VSMC abnormal proliferation and migration such as restenosis. We also should note here that the doses (10 mg/kg/day) of LiCl used *in vivo* in the present study are lower than that used in the clinic (20–40 mg/kg/day for acute mania, 10–30 mg/kg/day for maintenance) [22]. This will decrease the risk to toxicity and improve the patient tolerance in the future application. Indeed, the serum levels of hepatic injury hallmarks remained unchanged in our animals. We did not find any abnormality in food intake and activity as well (data not shown).

PGC-1α, initially identified as a PPARγ coactivator, regulates diverse metabolic programs through coactivating selective transcriptional factors associated with regulatory elements of target genes. Recently, It is reported that PGC-1α induces E-cadherin expression while decreasing motility of human hepatoma HepG_2_ cells [23]. PGC-1α also plays an important role in the regulation of genes responsible for the detoxification of ROS, providing an antioxidant defense against oxidative stress [24]. In the present study, we found that PGC-1α expression levels are negatively correlated with the rate of VSMC proliferation and migration, as well as ROS generation, which are coincided with previous findings and confirm the possibility that PGC-1α may serve as a promising drug target to regulate cardiovascular physiological homeostasis. More interesting, however, is the manner by which LiCl regulates PGC-1α levels. Our data suggest that the role of LiCl is mainly to increase PGC-1α protein rather than gene expression. How this occurs remains a subject of further investigation, but our experiments suggest that PGC-1α is not regulated on the level of protein stability. It is thus likely that other post-translational mechanisms, such as selective translation, may contribute.

Mitogenic signals such as serum activate diverse pathways that contribute to VSMC proliferation. Among which, the cell cycle is a common convergent point. Progression through several major checkpoints in the cell cycle is controlled by multiple protein kinases, each of which contains a regulatory cyclin component and a catalytic CDK [25]. The expression levels of each component, their phosphorylation status, and the presence of specific CDK inhibitors regulate the activity of these kinases. p21 and p27 are negative regulators of the protein kinases and cyclins, and can block the cell cycle at G_0_/G_1_ phase [26]. Here, we showed that treatment of LiCl caused cell cycle arrest, accompanied with the reduced expression levels of PCNA, cyclin D1/E and CDK 2/4/6. In contrast, the protein levels of p21 and p27 were correspondingly increased. Based on these findings, it is reasonable to speculate that the inhibitory effects of LiCl on VSMC proliferation are associated with the suppression of the whole cell cycle process.

As one of the feature events in the development of restenosis, VSMC migration is finely regulated by a series of complex mechanisms. When sensing mitogenic signals, VSMCs increase the gene expression levels of VEGF, a major pro-angiogenic factor enriched in human atherosclerotic plaques [27]. VEGF, once released, activates the downstream signaling cascades including the phosphorylation of FAK and the increase of eNOS expression in a paracrine manner through activating VEGF receptors [28]. These events will subsequently evoke migration-associated pathophysiological changes (MMP production and secretion, cytoskeletal rearrangement and increased vasopremeability). Here, the observations at the morphological and molecular levels confirm the inhibitory effects of LiCl on VSMC migration: 1. Would healing and transwell assays showed that LiCl inhibited serum-induced VSMC migration rate; 2. VEGF, phospho-FAK and eNOS expression levels were all down-regulated by LiCl; 3. MMP-2/9 expression levels and activities were reduced by LiCl. These findings also suggest that such effects are achieved at least partially through inhibiting VEGF signaling axis. On the other hand, our data showed that FBS and injury induced a rapid and sustained activation of GSK-3β (de-phosphorylation), but LiCl retarded GSK-3β activation in VSMCs as expected. As a multifunctional kinase, GSK-3β is ubiquitously expressed in eukaryotes, that inhibits cell proliferation and migration through limiting the availability of cyclin D1 [29], decreasing the expression of the CDK inhibitor p27^Kip1^ [30], and negatively modulating FAK catalytic activity [31]. Our findings seem to be discrepant to the current theory. One possible explanation is that LiCl plays versatile roles in regulating various transcriptional factors and cofactors (e.g. PGC-1α in the present study) in addition to inhibiting GSK-3β. Just as the two arms of a balance, although inhibition of GSK-3β by LiCl may cause the accelerated cell proliferation and migration, other negative factors evoked by LiCl simultaneously inhibit these processes in a more robust manner, leading to a negative net effect.

Chronic inflammation is a pathogenic feature of cardiovascular diseases mediated by substances including proinflammatory cytokines and free fatty acids [32]. This promotes generation of ROS in vessel cells and leads to the oxidative stress. Conversely, oxidative stress stimulates production of chemokines, cytokines and adhesion molecules as well as activation and proliferation of lymphocytes. Thus, oxidative stress and inflammation are involved in a self-perpetuating cycle, contributing importantly to the pathogenesis of cardiovascular diseases. In the present study, we showed that LiCl decreases ROS generation, as well as the expression of adhesion molecules (ICAM-1 and VCAM-1) and OPN, a chemotactic protein tightly associated with VSMC inflammation, calcification and migration. Similarly, the anti-oxidative and anti-inflammatory effects of this drug on glia cells [33], renal cells [34] and cerebral cortical cells [35] have also been reported. Therefore, such protection seems not to be cell type-specific and further evidence should be provided in a broader context and settings.

In conclusion, our findings demonstrate for the first time that LiCl inhibits VSMC proliferation and migration and alleviates balloon injury-induced neointima formation. Transcriptional coactivator PGC-1α plays an important role in mediating these beneficial effects (Fig. 7E). These findings imply that in addition to its current clinic use in the treatment of bipolar disorder, LiCl also has great potentials in the prevention and treatment of cardiovascular diseases related to VSMC abnormal proliferation and migration. On the other hand, our study confirms the possibility that PGC-1α may serve as a promising drug target to regulate cardiovascular physiological homeostasis.

## Materials and Methods

All animal experiments in this investigation were conducted in compliance with the Guide for the Care and Use of Laboratory Animals published by the US National Institutes of Health (NIH publication No. 85-23, revised 1996) and approved by the Animal Care and Use Committee of Nanjing Normal University, China (Permit Number: 2090658).

### Cell culture

VSMCs were isolated from the thoracic aortas of 3- to 4-week-old male Sprague-Dawley rats as described previously [36]. Cells at passages 4–8 were used in experiments. To knock down PGC-1α expression levels, confluent VSMCs were infected with recombinant adenoviruses expressing PGC-1α siRNA or scrambled siRNA (kindly provided by Dr. Jiandie D. Lin, Life Sciences Institute, University of Michigan) at the MOI of 50 for 30 hr. For drug treatment, serum-starved VSMCs were pretreated with LiCl (Sigma, 5, 10, 15 mmol/L) for 30 min and then challenged with 10% fetal bovine serum (FBS) (Invitrogen), 0.2 mmol/L BSA-conjugated oleic acid (Sigma), or 10 ng/mL PDGF-BB (Sigma), in the presence of LiCl for 24 hr unless otherwise indicated. Control cells were treated with 15 mmol/L NaCl (Sigma).

### RT-qPCR

Total RNA from VSMCs was extracted using Trizol reagent (Invitrogen, Carlsbad, USA). Two micrograms of total RNA was reverse-transcribed into complementary DNA. 18 s ribosomal RNA served as an internal control for total complementary DNA content. mRNA levels were quantified by real-time RT-PCR using SYBRGreen Master Mix (Applied Biosystems, Foster City, USA). Samples were amplified using the Mastercycler ep realplex2 system (Eppendorf, Hamburg, Germany). Primer sequences were: 5′- ATGGTTTCATTACCTACCGTTACAC-3′ (F) and 5′- AAGCAGGGTCAAAATCGTCTGAGT-3′ (R) for PGC-1α; 5′-AAACGGCTACCACATCCAAG-3′ (F) and 5′- CCTCCAATGGATCCTCGTTA-3′ (R) for 18 s rRNA.

### Western blot

VSMCs were lysed in RIPA buffer containing 50 mmol/L pH 8.0 Tris-HCl, 150 mmol/L NaCl, 1% NP-40, 1% sodium deoxycholate, 0.1% sodium dodecylsulfate, 0.1 mmol/L dithiothreitol, 0.05 mmol/L phenylmethyl-sufonylfluoride, 0.002 mg/mL aprotinin, 0.002 mg/mL leupeptin, and 1 mmol/L NaVO_3_. Dc protein assay reagent (Bio-Rad, Hercules, CA) was used to quantify the protein concentration. We use 10% SDS-PAGE to loaded and separate proteins. Proteins were transferred onto polyvinylidene difluoride membranes (Millipore, USA). The membranes were incubated with appropriate primary antibodies overnight. HRP-conjugated secondary antibodies (Santa Cruz) were applied to visualize and bound to primary antibodies. For the antibody information, the antibodies against cyclin E, PCNA, CDK2, cyclin D1, CDK6, p27, GSK-3β, phospho-GSK-3β, FAK, and phospho-FAK were purchased from Cell Signaling Technology, the antibodies against CDK4 and p21 were purchase from BD Biosciences, the antibodies against ICAM-1, VCAM-1, OPN, MMP-2, MMP-9, eNOS, VEGF and GAPDH were purchased from Santa Cruz.

### Determination of PGC-1α protein degradation

To explore how LiCl regulates PGC-1α protein expression, the cycloheximide (CHX) chase assay and coimmunopreciptation assay targeting PGC-1α ubiquitination were applied. For the CHX chase, VSMCs were incubated in medium containing 100 µg/ml CHX (sigma) and harvested at the indicated time points. The cells were lysed and subjected to Western blot. For the ubiquitination assay, VSMCs were lysed in RIPA buffer and spun at 13,000 rpm at 4°C for 10 min to remove cellular debris. Coimmunopreciptation was performed by incubation with polyclonal anti-PGC-1α antibody (Santa Cruz) overnight. Pre-cleared protein A/G PLUS-Agarose beads (Santa cruz) were then added and incubated for additional 4 hr. The beads were intensively washed and boiled at 100°C with sample buffer for 5 min. The immunoprecipitates were analyzed by Western blot with monoclonal anti-ubiquitin antibody (Santa cruz).

### Immunocytochemistry

VSMCs were fixed in ice-cold 4% paraformaldehyde for 30 min followed by the blocking in 5% goat serum for 1 hr. Cells were then incubated with rabbit polyclonal anti-PGC-1α antibody overnight at 4°C. After repeated washing, cells were incubated with HRP-conjugated secondary antibody. DAPI staining for nuclear localization was performed simultaneously. Images were captured using a Nikon fluorescence microscope (Ti-S 533665).

### Cell proliferation assay

Cell proliferation was analyzed by using MTT and EdU incorporation assays. For MTT measurement, VSMCs were seeded in 96-well plates (1×10^4^ cells per well) and then subjected to indicated treatments. After that, MTT (0.2 mg/mL) was added to each well and incubated for 4 hr. The supernatant was removed and the formazan crystals were dissolved in DMSO. Cell proliferation was assessed by measuring the absorbance at 550 nm using a microplate reader. We also measured DNA synthesis by EdU incorporation method as described previously. In brief, VSMCs were incubated with EdU (10 µmol/L) for 2 hr and fixed with 4% paraformaldehyde. The cells were doubly stained with anti- EdU primary antibody (Promega) and DAPI. The percentages of Edu-incorporated cells were calculated.

### Cell migration assay

Cell migration was analyzed by using wound healing and transwell assays. For the wound healing assay, VSMCs were seeded in 6-well plates (1.5×10^5^ cells per well) and grew to confluence. 24 hr after serum deprivation, the cells were subjected to virus infection and LiCl pretreatment as indicated. To eliminate the possibility that the cell migration is dependent on proliferation, we next treated the cells with mitomycin C (20 µmol/L), a potent DNA crosslinker, for 2 hr and mounted cells to a reusable template to create a standard wound (<3 mm) (This time point was set as 0 hr). Later, 10% FBS was added to the cells and incubated for 24 hr in the presence of LiCl. Wound closure rates were followed with a reference point in the field of the wound at the bottom of the plate by direct microscopic visualization. The procedure permitted photographing the identical spot each time. The remaining cell-free area was determined via microphotography and performed immediately after 24 hr injury. For the transwell assay, a 24-well modified Boyden chamber containing fibronectin-coated polycarbonate membranes (8 µm pore-size, BD Bioscience, USA) was used. Briefly, the lower wells of the chamber were filled with phenol red-free DMEM supplemented with/without 10% FBS in the presence or absence of LiCl, as indicated above. The filters were coated with 50 mg/mL fibronectin and fixed atop the bottom wells. 1×10^5^ per well VSMCs were allowed to migrate for 6 hr and non-migrated cells were removed from the upper side of the membrane with cotton swabs. Cells on the lower side of the membrane were stained with Hoechst 33342, and then counted in five randomly selected squares per well with a fluorescence microscope (Nikon, Japan, Ti-S 533665). Data was presented as numbers of migrated cells per field.

### Measurement of ROS generation

ROS generation in VSMCs was measured by loading cells with MitoTracker Red probe (CM-H2XRos) (10 µmol/L) for 30 min at 37°C. Cells were then rinsed twice with PBS, and the cultures were photographed with a fluorescence microscope (Nikon, Japan, Ti-S 533665).

### Flow cytometry

VSMCs were fixed in 70% ethanol, washed twice with PBS, and stained with a 50 µg/mL propidium iodide (PI) solution containing 0.1% Triton X-100, 0.1 mmol/L EDTA, and 50 µg/mL RNase A. Fluorescence was measured and analyzed using a FACSCalibur flow cytometer (Becton Dickinson Immunocytometry Systems).

### ELISA

Culture medium was collected and centrifuged at 2000 rpm for 10 min at 4°C. The concentration of MMP-2 and MMP-9 in the supernatant was measured by using commercial ELISA kits based on the principle of double-antibody sandwich technique.

### Measurement of NO levels

The culture medium was collected and centrifuged at 4,000 g for 10 min. The nitrate/nitrite levels in the supernatants was measured by using a commercial kit (Jiancheng, Nanjing, China) according to the to the manufacturer's protocol.

### Animal experiments

All surgery was performed under sodium pentobarbital anesthesia, and all efforts were made to minimize suffering. Male Sprague-Dawley rats weighing 350 to 400 g underwent guidewire endothelial denudation injuries by the insertion of a balloon catheter of 1.5-mm diameter (Medtronic, Minneapolis, USA) through the left external carotid artery and advanced 4 cm toward the thoracic aorta. The balloon was distended and then pulled back to the bifurcation with constant rotation. This procedure was repeated 2 more times to ensure complete endothelial denudation. Ipsilateral carotid arteries underwent a similar operation without injury and served as sham controls. To knockdown PGC-1α expression *in vivo*, 15 µg of the siRNA against PGC-1α (Invitrogen) dissolved in 30% pluronic gel solution was perivascularly delivered to the rat carotid arteries immediately after injury as described previously [37]. A scramble Stealth RNAi duplex (catalog no. 12935, Invitrogen) served as a negative control.

From the second day after surgery, rats in lithium-treated group received LiCl (10 mg/kg/day) in tap water by gavage for a period of 14 consecutive days. Control rats received equal amount of sodium chloride solution. On the day 15, the blood flow volume and the inner diameter of left external carotid artery were analysed by using a doppler ultrasound instrument (VisualSonics Vevo2100).

### Morphometric analysis & Immunohistochemistry

The rats were sacrificed and the left carotid artery was isolated, fixed in 4% paraformaldehyde solution for 24 hr in situ, processed for paraffin embedding, and cut into 4 µm transverse sections. The tissue sections were either subjected to routine H&E and Masson-Goldner trichrome staining for the detection of collagenous fibers and muscle fibers or incubated with indicated primary antibodies at 4°C overnight for the later immunostaining by using diaminobenzidine (DAB). Non-immune IgG was used as a negative control. Six cross-sectional areas of various blood vessel layers (the lumen, intima and media) were randomly selected and measured with a computer-based Image Pro Plus Analyzer 4.5 (Media Cybernetics) system. The intima-to-media thickness ratio (I/M ratio) was calculated from the means of these determinations.

### Serological analysis

Blood was collected in non-heparinized tubes and centrifuged at 3000 rpm for 10 min at 4°C. The serum levels of lithium was detremined by using L-PAD (Large Programmable Array Detector). Each measurement was repeated for six times and the mean concentrations were calculated. Serum levels of alanine aminotranferease (ALT), aspartate aminotransferase (AST), alkaline phosphatase (ALP), γ-glutamyltransferase (GGT) and lactate dehydrogenase (LDH) were measured by using a SELECTRA JUNIOR Version 04 autoanalyzer (Vital Scientific, Spankeren, The Netherlands).

### Data analysis

Groups of data are presented as mean ± standard error. Data were analyzed using one-way ANOVA followed by Fisher's LSD post-hoc test. Calculations were performed using SPSS for Windows version 12.5S statistical package (SPSS, Chicago, USA). A value of *P*<0.05 was considered statistically significant.

## Supporting Information

Figure S1 **The upregulation of PGC-1α protein expression by LiCl is mitogen-independent.** Cells were pretreated with 15 mmol/L LiCl for 30 min and then stimulated with 0.2 mmol/L BSA-conjugated oleic acid (OA) or 10 ng/mL PDGF-BB (dissolved in DMSO) for 24 hr in the presence of LiCl. Cells were then lysed and subjected to Western Blot to detect PGC-1α protein expression.
(TIF)

Figure S2 **Validation of the knockdown efficiency of siRNA adenoviruses against PGC-1α.** Cells were treated as previously described in Fig. 2 and PGC-1α expression levels were determined by RT-qPCR (A) and Western blot (B). GAPDH was used as an internal control. Data are presented as means ± SEM from three independent experiments. ^*^ *P*<0.05 compared with the control siRNA group.
(TIF)

Figure S3 **LiCl increases PGC-1α protein expression and GSK-3β phosphorylation levels** ***in vivo*** **.** Balloon-induced injury and siRNA transfection were performed in rat carotid arteries, followed by LiCl treatment for 14 days. PGC-1α protein expression and GSK-3β phosphorylation levels in VSMC layer were determined by Western blot. A representative blot was shown.
(TIF)

Figure S4 **PGC-1α silencing blocks LiCl-induced alleviation of neointimal hyperplasia.** (A) Representative cross-sections of H & E stained carotid arteries treated with PGC-1α siRNA or scramble siRNA, plus LiCl treatment for 14 days after injury. The scale bar indicates 200 µm. (B) I/M thickness ratio analysis. ^**^ *P*<0.01 compared with the control group; ^++^ *P*<0.01 compared with the serum-treated group; ^##^ *P*<0.01 compared with the control siRNA group.
(TIF)
