# Supplementary figures and images for: Lithium Chloride Inhibits Vascular Smooth Muscle Cell Proliferation and Migration and Alleviates Injury-Induced Neointimal Hyperplasia via Induction of PGC-1α
Source: PLoS One. 2013 Jan 31;8(1):e55471. doi: 10.1371/journal.pone.0055471 (PMC3561220; doi:10.1371/journal.pone.0055471)

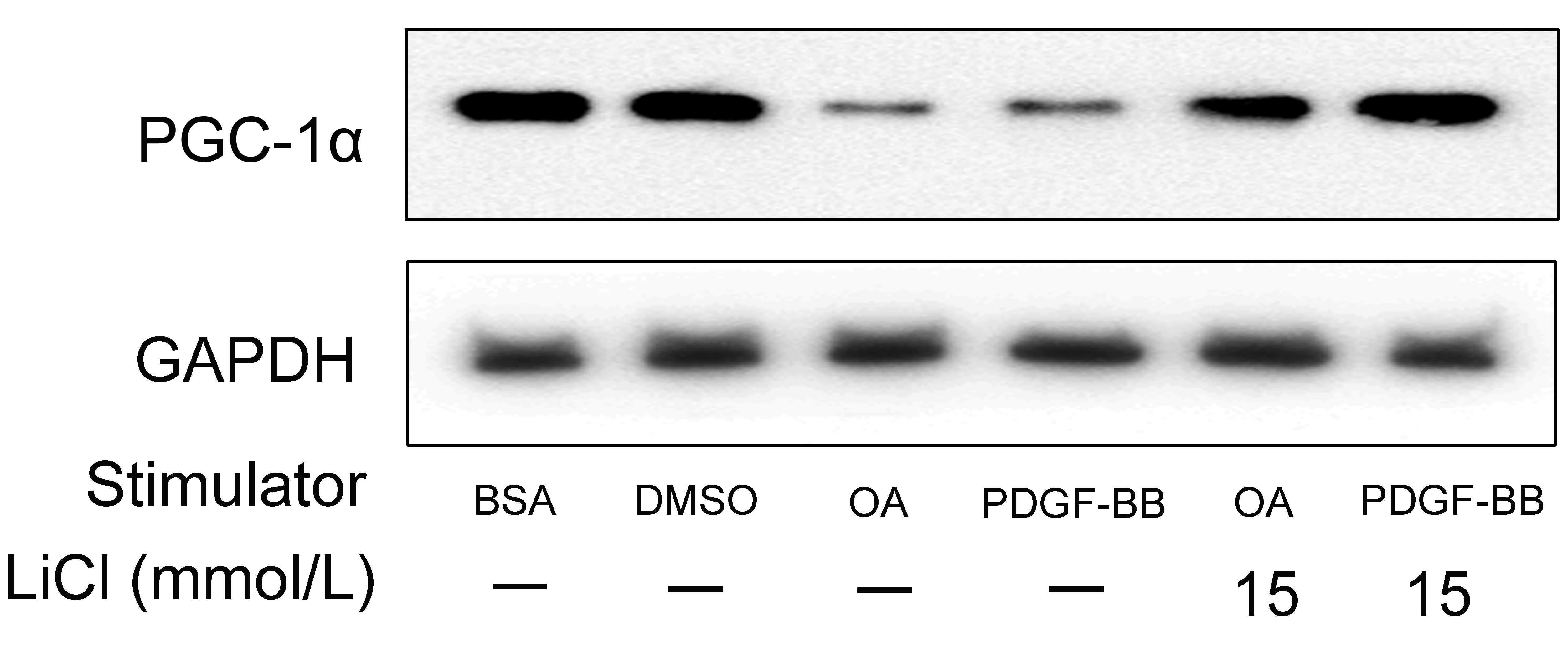

Supplement: Figure S1 — The upregulation of PGC-1α protein expression by LiCl is mitogen-independent. Cells were pretreated with 15 mmol/L LiCl for 30 min and then stimulated with 0.2 mmol/L BSA-conjugated oleic acid (OA) or 10 ng/mL PDGF-BB (dissolved in DMSO) for 24 hr in the presence of LiCl. Cells were then lysed and subjected to Western Blot to detect PGC-1α protein expression. (TIF) [file pone.0055471.s001.tif]

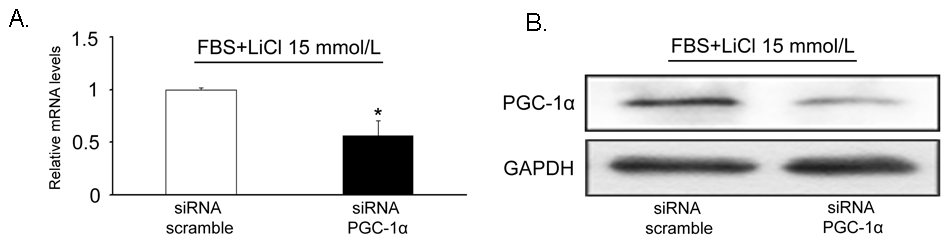

Supplement: Figure S2 — Validation of the knockdown efficiency of siRNA adenoviruses against PGC-1α. Cells were treated as previously described in Fig. 2 and PGC-1α expression levels were determined by RT-qPCR (A) and Western blot (B). GAPDH was used as an internal control. Data are presented as means ± SEM from three independent experiments. * P<0.05 compared with the control siRNA group. (TIF) [file pone.0055471.s002.tif]

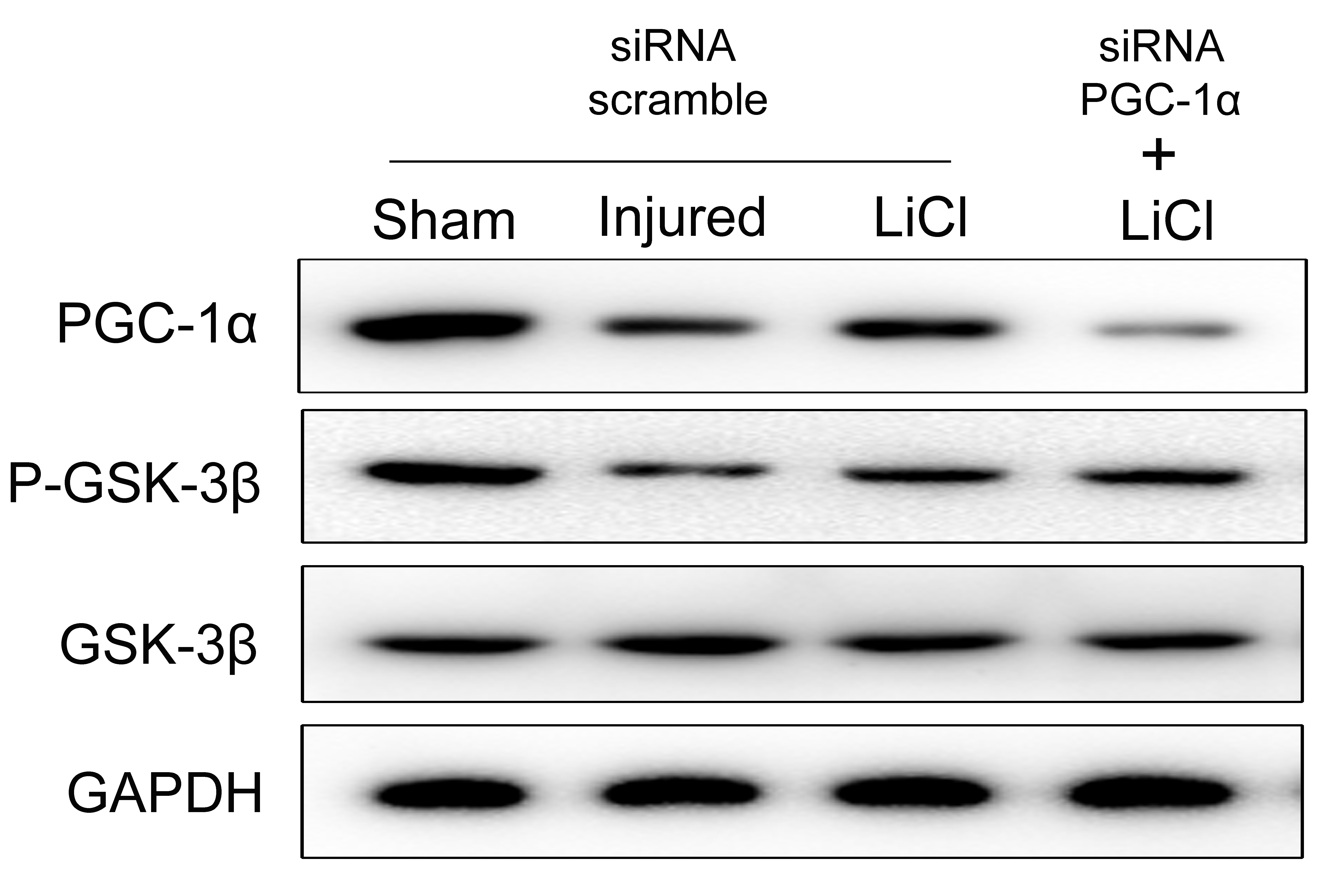

Supplement: Figure S3 — LiCl increases PGC-1α protein expression and GSK-3β phosphorylation levels in vivo . Balloon-induced injury and siRNA transfection were performed in rat carotid arteries, followed by LiCl treatment for 14 days. PGC-1α protein expression and GSK-3β phosphorylation levels in VSMC layer were determined by Western blot. A representative blot was shown. (TIF) [file pone.0055471.s003.tif]

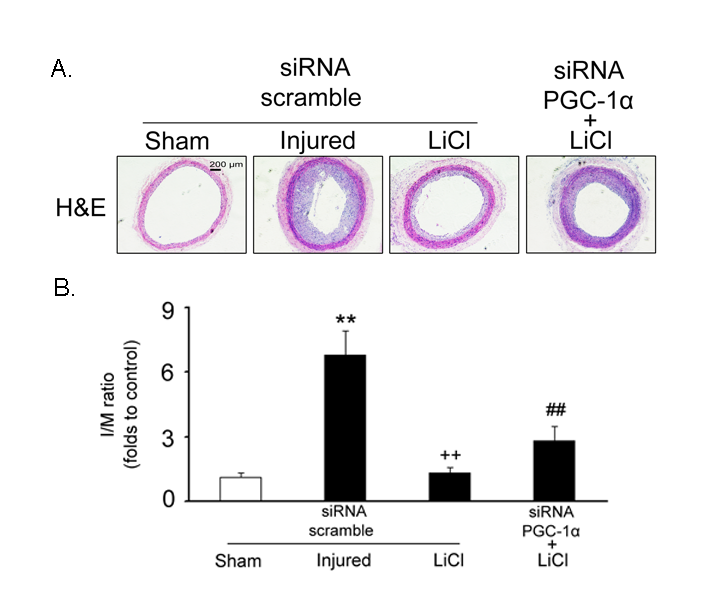

Supplement: Figure S4 — PGC-1α silencing blocks LiCl-induced alleviation of neointimal hyperplasia. (A) Representative cross-sections of H & E stained carotid arteries treated with PGC-1α siRNA or scramble siRNA, plus LiCl treatment for 14 days after injury. The scale bar indicates 200 µm. (B) I/M thickness ratio analysis. ** P<0.01 compared with the control group; ++ P<0.01 compared with the serum-treated group; ## P<0.01 compared with the control siRNA group. (TIF) [file pone.0055471.s004.tif]
